# The Case of the Children's Hospitals

**Published:** 1919-02-08

**Authors:** Arthur Lucas, John Murray

**Affiliations:** Chairman. The Hospital for Sick Children, Great Ormond Street, W.C.; Vice-Chairman. The Hospital for Sick Children, Great Ormond Street, W.C.


*To the Editor of* The Hospital.

### The Case of the Children's Hospitals.

*To the Editor of* The Hospital.

Sir,—We have read in The Hospital for February 1 the report of the discussion which took place at the Royal Society of Medicine on the Draft Bill for the College of Nursing, and we beg to protest against the way in which Children's Nursing has been dealt with in that debate, as in all the negotiations which have taken place during the formation of the said College of Nursing.

In the first place we find the Chairman, Sir Cooper Perry, referring to Children's Hospitals as "Special Hospitals," and dismissing them summarily from all participation in the benefits of the Bill.

Surely these gentlemen must be fully aware that such hospitals as Great Ormond Street are not special hospitals, but general hospitals with an age limit.

One of the speakers said, "In the military hospitals they have been taught nothing of, say, early conditions, such as rickets or the onset of malnutrition—all those conditions of illness which commence so insidiously," but we would ask, can these be better studied than in a children's hospital?

The speakers appear to be unaware of the fact that almost all the diseases and injuries dealt with in a general hospital are also dealt with in a good children's hospital, and the helplessness of a large majority of the patients calls for greater skill and more thorough training on the part of the nurses.

At no stage in the promotion of this Bill have children's hospitals been adequately represented, or has their case been properly put forward.

The whole question cannot be discussed in such a letter as this, but we can claim to have had forty-three and thirty-three years respectively of incessant and close experience of children's hospital work, and we assert that it is much easier for a well-trained child's nurse to learn what is necessary of adult training than *vice versâ*.—We are, Sir, your obedient servants,

Arthur Lucas, Chairman.
John Murray, Vice-Chairman.

The Hospital for Sick Children,
Great Ormond Street, W.C.
February 4, 1919.

